# Patient-centered approach to the management of drug-resistant tuberculosis in France: How far off the mark are we?

**DOI:** 10.1371/journal.pgph.0000313

**Published:** 2022-04-20

**Authors:** Yousra Kherabi, Bastien Mollo, Sandrine Gerard, François-Xavier Lescure, Christophe Rioux, Yazdan Yazdanpanah

**Affiliations:** 1 Service des Maladies Infectieuses et Tropicales, Centre Hospitalier Universitaire Bichat-Claude Bernard, Paris, France; 2 Inserm U1137, IAME, Université de Paris, Paris, France; Universidad Norbert Wiener, PERU

## Abstract

Drug-resistant tuberculosis (DR-TB) is a major public health concern worldwide. The prolonged isolation required is a source of challenges for both healthcare workers and patients, especially in high-income countries where DR-TB patients are frequently migrants with vulnerabilities. However, data on the needs of these vulnerable patients are scarce. Our objective was to identify and quantify conflict or inappropriate care situations experienced by both DR-TB patients and healthcare workers. This 10-year retrospective observational study (01/2008 to 10/2018) was conducted in a referral center for resistant tuberculosis management in Paris, France. Sixty-five DR-TB patients were hospitalized during the study period. Their demographic, clinical and social characteristics and any conflict or inappropriate care situations they experienced with healthcare workers while hospitalized were analyzed. Conflict or inappropriate care situations with healthcare workers were reported for 24 patients during their stay (36.9%). Eleven patients (16.9%) had difficulty adhering to respiratory isolation rules, 15 (23.1%) were discharged against medical advice, 9 (13.8%) were excluded from hospital for disciplinary reasons, verbal or physical violence was reported for 7 patients (10.8%), and 4 arrests (6.2%) were made by the police. Conflict situations were reported more often when there was a language barrier (70.8%, p<0.0001). More than one-third of patients with DR-TB in this referral center experienced at least one inappropriate care situation with healthcare workers. This study illustrates the urgent need to promote a patient-centered approach and to respond to the challenges of its practical implementation.

## Introduction

Multidrug-resistant tuberculosis (MDR-TB) and extensively drug-resistant tuberculosis (XDR-TB) are major public health concerns worldwide: in 2019, there were 465,000 incident cases of rifampicin-resistant tuberculosis (RR-TB), of which 78% were MDR-TB [1, 2]. It was estimated that 10% (46,000) of the global RR/MDR-TB pulmonary cases in 2019 were notified in Europe [3] however the proportion of RR/MDR-TB among TB cases in the WHO European region substantially outpace the global average [2, 3]. In France, the annual number of MDR-TB patients was stable at around 50 between 2006 and 2010, but dramatically increased subsequently [4] and reached more than 100 patients in 2014 [5].

Drug-resistant TB (DR-TB) is associated with high morbidity and mortality (up to 40% mortality in MDR-TB and 60–70% in XDR-TB in some endemic countries each year) [6]. MDR and XDR-TB are known to be difficult and long to treat: though shorter regimens are tending to be more frequently used [7–9], a 12- to 24-month antibiotic treatment is recommended for most patients with an unsatisfying success rate of 54% for MDR-TB and 34% for XDR-TB [10]. These diseases are also known to be a major threat to the healthcare workforce through nosocomial transmission [11]. Moreover, both patients and healthcare workers face behavioral challenges as a result of the required prolonged isolation; especially in high-income countries where the DR-TB population frequently comprises migrants [12] with vulnerabilities [13]. However, the literature data regarding these issues are scarce for patients with DR-TB [14]. Recent WHO guidelines on DR-TB treatment emphasize the urgent need to address the challenge of a patient-centered approach [7], defined as a health-care model in which patients are “served by and are able to participate in trusted health systems that respond to their needs in humane and holistic ways” [15]. Nevertheless, data on conflict situations between patients and healthcare workers during hospitalization, reflecting a mismatch between patients’ expectations and care provided, are lacking. The objective of this study was to describe qualitatively and quantitatively the conflict or inappropriate care situations reported during hospitalization, from the perspective of healthcare workers, in a high-income country.

## Patients and methods

### Study design

We conducted an observational retrospective study over 10 years (01/01/2008 to 10/01/2018) in a referral hospital for resistant tuberculosis management in Paris, France, namely Bichat Claude Bernard Hospital.

All patients with MDR/XDR TB hospitalized during this time period, identified either through hospital or laboratory database searches, were included. MDR-TB was then defined by bacillary resistance to rifampicin and isoniazid, XDR-TB as MDR-TB combined with resistance to a fluoroquinolone and at least one second-line injectable agent (amikacin, capreomycin or kanamycin) (1). At that time, preXDR-TB was unofficially defined as MDR-TB combined with either a fluoroquinolone or at least one second-line injectable aminoglycoside. Of note, during the study period, treatment regimens were designed in accordance with WHO treatment guidelines and in consultation with the French MDR-TB Consilium, a multidisciplinary team coordinated by French National Reference Center for Mycobacteria (Paris, France) [7, 16–18]. Patients were isolated in hospital until sustained sputum culture conversion was obtained, defined as two consecutive negative cultures, collected at least 30 days apart [16]. Patients could be visited, by one person at a time. Of note, psychological counseling was routinely proposed to patients who report a difficult life event (such as a migratory journey) at admission, or after a possible mental health issue or a conflict situation was spotted by a health-care worker taking care of the patient.

We retrospectively analyzed the fully-anonymized charts of every patient, including medical records, and everyday nurse reports in which patients’ behavior is systematically described at least twice a day. Medical records included all outpatient consultation reports and medical charts during hospitalization in our center and other collaborating centers e.g. sanatorium and surgical reports. A standardized form was used to screen the charts.

### Outcome measures

The primary composite outcome was the report of a conflict or inappropriate care situation, defined as the occurrence of at least one of the following situations of non-compliance to our department rules: repeated non-compliance with isolation rules, as defined by the WHO [7]; leaving the medical department without medical authorization; discharge against medical advice; repeated refusal of at least one anti-TB treatment without reason by a patient despite repeated explanations by at least two different healthcare workers; repeated refusal of blood test or procedure without reason despite repeated explanations by at least two different healthcare workers; alcohol or drug consumption during hospitalization; leaving the country without family or medical justification; verbal violence against staff (defined as insults and/or threats); physical violence against staff (defined as assault and battery); stealing during hospitalization; legal custody during hospitalization; imprisonment during TB care; and/or exclusion from the department for disciplinary reasons (i.e. exclusion after a multidisciplinary meeting because the patient repeatedly experienced the situations described above, leading to care failure and staff exhaustion).

We also collected data on the clinical, demographic and social characteristics of this population, to analyze the links between patients’ backgrounds and conflict situations.

Of note, irregular migration and asylum seeking were defined following the European Union Law [19].

### Statistical analyses

Categorical variables are expressed as number (%) and compared using Fisher test. Continuous variables are expressed as median [interquartile range (IQR)], first assessed for normality and then compared with the non-parametric Wilcoxon test. Univariate analyses were performed with a binary logistic regression. A *P* value < 0.05 was considered significant. All statistical tests were performed with STATA.14 software (Copyright 1985–2015 StataCorp LP, StataCorp, 4905 Lakeway Drive, College Station, Texas 77845 USA).

### Ethical approval

The study protocol was approved by the ethics evaluation committee of INSERM, the Institutional Review Board of the French Institute of Medical Research and Health (avis n°18–541). According to Loi Jardé, written consent was not required but a letter of information was sent to all participants in their native language.

## Results

Sixty-six patients with MDR/XDR TB were identified to have been hospitalized in our hospital during the study period. One patient was excluded because no complete nursing record was found. The data of 65 patients were analyzed.

### Patient characteristics

Forty-nine out of 65 patients were men (75.4%), with a median age of 35 years. Sixty out of 65 were born outside France (92.3%) and 24 out of 65 (36.9%) had a language barrier, defined as the non-comprehension of the three major languages that were spoken by staff members (French, English and Spanish) (**Table 1**). Most of our patients had a relevant social situation: 41 of them were irregular migrants or asylum-seekers (63.1%) and 27 were homeless (41.5%). Fifty-one (78.5%) patients had contagious TB and were isolated for a median of 180 days (interquartile range [132–251], data available for 50 patients). Two (3.1%) patients died from tuberculosis, one from meningeal tuberculosis, and one from respiratory failure in advanced respiratory tuberculosis. Thirty (46.2%) patients completed their follow-up and all of them were considered cured for TB. Eighteen patients (27.7%) were lost to follow-up. Fourteen (21.5%) patients were still being actively treated at the time of data collection. For three (4.6%) patients, TB outcome was not evaluated because they moved to another area in France.

**Table 1:** Characteristics of 65 patients with MDR/XDR TB in a referral center in France, 2008–2018.

Characteristic	Value
Age, years [IQR*]	35 [28–41]
**Male, no. of patients (%)**	49 (75.4)
**Area of origin, no. of patients (%)**	
Africa	27 (41.5)
Eastern Europe	24 (36.9)
France	6 (9.2)
Asia	5 (7.7)
South America	3 (4.6)
**Language barrier, no. of patients (%)**	24 (36.9)
**Social situation, no. of patients (%)**	
Homeless	27 (41.5)
Past imprisonment	11 (16.9)
Past or present intravenous drug use	9 (13.8)
Sex work	3 (4.6)
Irregular migrant or asylum-seeker	41 (63.1)
**Comorbidities, no. of patients (%)**	
Previous anti-TB treatment for current episode	26 (40.0)
HIV infection	15 (23.1)
Hepatitis C infection	7 (10.8)
Hepatitis B infection	5 (7.7)
**Time period between arrival in France and first consultation, no. of patients (%)**	
< 1 day	12 (18.5)
1 day-1 month	12 (18.5)
1 month- 6 months	3 (4.6)
> 6 months	36 (55.4)
Unknown	2 (3.1)
**Resistances, no. of patients (%)**	
MDR	43 (66.2)
PreXDR	12 (18.5)
XDR	10 (15.4)
**Smear positive, no. of patients (%)**	46 (70.8)
**Organs involved, no. of patients (%)**	
Lungs	51 (78.5)
Disseminated	9 (13.8)
Others	5 (7.7)
**Death during TB-care hospitalization, no. of patients (%)**	3 (4.6)
Death due to TB	2 (3.1)
Death due to other cause	1 (1.5)

*IQR: interquartile range.

### Outcomes

Twenty-four patients experienced at least one conflict or inappropriate care situation with healthcare workers during their stay (36.9%) (**Table 2**). Among them, all at least once experienced difficulty adhering to medical prescription of isolation and/or drugs and/or complementary examinations and/or tests. Among the 11 patients (16.9%) who did not follow isolation rules, 1 patient needed to be guarded by a policeman in order to prevent him from leaving his hospital room without medical authorization. Four patients left the facilities and went from France to Germany (6.2%) without notice, 2 of them while they were hospitalized and still contagious (3.1%). Seven patients were verbally and/or physically violent against staff (10.8%). A physician and an assistant nurse filed a complaint to the police department against one of these seven patients for assault and battery. One patient was caught in the act of theft during his stay, 2 patients were brought back by policemen as they were reported stealing outside of the hospital. Two patients were imprisoned while they were no longer contagious, but they still needed treatment in jail. Treatment was optimally conducted in prison, according to the patient’s treatment protocol. One patient was imprisoned during outpatient follow-up, as TB treatment was already finished.

**Table 2:** Description of conflict situations during tuberculosis management, in 65 patients with MDR/XDR TB in a referral center in France, 2008–2018.

Characteristic	Value
**Medical care non-adherence, no. of patients (%)**	24 (36.9)
Non-respect of respiratory isolation rules	11 (16.9)
*Need for 24/24 watch*	1 (1.5)
Leaving the medical department (>12 h)	13 (20.0)
Discharge against medical advice	4 (6.2)
Refusal of at least one treatment	9 (13.8)
Refusal of complementary exams	8 (12.3)
Exclusion from department for disciplinary reasons	9 (13.8)
Alcohol or cannabis consumption during hospitalization	5 (7.7)
Departure to another country without family/medical justification	5 (7.7)
*Germany*	4 (6.2)
*Switzerland*	1 (1.5)
**Violence, no. of patients (%)**	7 (10.8)
Physical violence	2 (3.1)
*Complaint lodged by staff*	1 (1.5)
Verbal violence	7 (10.8)
**Altercation with the police, no. of patients (%)**	7 (10.8)
Theft during hospitalization	3 (4.6)
Detained in police custody during hospitalization	4 (6.2)
Imprisonment during TB care	3 (4.6)
*Treatment pursued in jail*	2 (3.1)

Patients with a language barrier experienced conflict or inappropriate care situations with healthcare workers more frequently than patients who spoke French, English and/or Spanish (70.8% compared to 17.1%, p<0.0001) (**Table 3**). Patients who had difficulty adhering to department rules were more often homeless (75.0% compared to 22.0%, p<0.0001), and irregular migrants or asylum-seekers (83.3% compared to 51.2%, p = 0.02). All nine patients (13.8%) with past or present intravenous drug use experienced dispute situations with staff. XDR-TB was not significantly more frequent in the “conflict situations” patient group than in patients without conflict (25.0% compared to 9.8%, p = 0.10), and patients in this group were more likely to have received a previous anti-TB treatment for the current episode (70.8% compared to 22.0%, p<0.001). Eleven patients had a history of imprisonment in their country of origin, all of them for declared political and/or war reasons. Ten of those patients (90.1%) had a conflict with healthcare workers.

**Table 3:** Comparison of patients who presented at least one conflict situation versus patients who did not, in 65 patients with MDR/XDR TB in a referral center in France, 2008–2018.

Characteristic	Patients without conflict situations: 41 patients	Patients with conflict situations: 24 patients	P-value^$^	OR [95% CI]^†^
Age, years [IQR*]	**35 [28–47]**	**33 [29–36]**	0.08	-
**Male, no. of patients (%)**	**28 (68.3)**	**21 (87.5)**	0.13	3.53 [0.89–13.99]
**Language barrier, no. of patients (%)**	**7 (17.1)**	**17 (70.8)**	<0.0001	10.02 [3.09–32.17]
**Social situation, no. of patients (%)**				
Homeless	9 (22.0)	18 (75.0)	<0.0001	8.86 [2.79–27.81]
Past imprisonment	1 (2.4)	10 (41.7)	<0.0001	24.6 [2.81–209.9]
Past or present intravenous drug use	0 (0)	9 (37.5)	<0.0001	NC^§^
Sex work	2 (4.9)	1 (4.2)	1	0.79 [0.07–9.21]
Irregular migrant or asylum-seeker	21 (51.2)	20 (83.3)	0.02	3.61 [1.13–11.54]
**Comorbidities, no. of patients (%)**				
Previous anti-TB treatment for current episode	9 (22.0)	17 (70.8)	<0.001	7.07 [2.28–21.75]
HIV infection	11 (26.8)	4 (16.7)	0.54	0.50 [0.14–1.82]
Hepatitis C infection	0 (0)	7 (29.2)	<0.001	NC
Hepatitis B infection	2 (4.9)	3 (12.5)	0.35	2.57 [0.39–16.6]
**Resistances, no. of patients (%)**			0.10	
MDR	31 (75.6)	12 (50.0)		Ref.^£^
PreXDR	6 (14.6)	6 (25.0)		3.58 [0.96–13.73]
XDR	4 (9.8)	6 (25.0)		3.81 [0.92–16.26]

*IQR: interquartile range.

^$^P-value: obtained by Fisher test for categorical variables and by Wilcoxon test for continuous variables.

^†^OR [IC 95%]: Odds ratio [95%-confidence interval].

^§^NC: not calculable.

^£^Ref.: reference category.

Nineteen patients (79.2%) who experienced a conflict situation with healthcare workers received psychological counseling, after the first altercation broke out, with a psychologist specialized in infectious diseases patients helped by a professional interpreter called by phone. All of those 19 patients did not refer to a conflict with a particular healthcare worker but rather with “the department rules”. Seventeen out of these 19 patients (89.5%) declared that “their main problem” was “isolation”. Of note, the 5 patients in the “conflict situation” group who did not undergo psychological counseling are the 5 patients who left the department to go to a foreign country.

## Discussion

Over 10 years in a referral center for drug-resistant tuberculosis, conflict or inappropriate care situations with healthcare workers concerned more than one-third of patients. This is to our knowledge the first study to provide quantitative and qualitative information on mismatches between expectations of patients and healthcare workers in DR-TB management during hospitalization. Indeed, the literature contains few case reports on severe incidents leading to serious consequences, such as community transmission due to non-compliance with isolation precautions [20].

These new data take all aspects of hospitalization in TB care into account, including sanatorium stays, hospitalization in the surgery department, etc. and shed light on the psycho-social issues of the management of DR TB in a high-income country. The WHO 2020 consolidated guidelines on DR-TB reveal several gaps in current knowledge in DR-TB treatment and care, especially in patient support best suited to vulnerable populations, such as migrants [7].

In our population, 60 patients (92.3%) were born outside France, 24 (36.9%) of them in Eastern Europe. Reported surveillance data in Europe suggest that 73.4% of MDR-TB cases are in patients born outside the reporting country, among whom 51.7% of MDR-TB cases occur in migrants originating from the EU [21]. Between 2007 and 2012, migrants accounted for 89.2% of MDR-TB infections in France (94% in Germany and 90.4% in UK), and 58.8% of all TB cases (58.7% in Germany and 69.1% in UK) [22].

It is well known that migrants are subjected to specific stressful experiences including racial discrimination, urban violence and abuse, forced removal or separation from their families, reclusion or deportation [23, 24]. In some studies, differences in resident status appear to be specifically associated with mental health outcomes and behavioral issues: anxiety and depression are more frequently reported by asylum-seekers and illegal migrants compared to labor migrants and residents [23]. Moreover, past detention is an additional factor for anxiety and behavioral disorders [25, 26]. In our series, 11 patients had a history of imprisonment, all of them for declared political and/or war reasons in their country of birth.

Mental health professionals are not trained to recognize and provide appropriate care for posttraumatic and/or stress-related disorders among migrants [24–27] and DR-TB treatment programs do not sufficiently consider comorbid mental disorders [28]. In our series, only 21 patients out of 41 irregular migrants and/or asylum-seekers received regular psychological support, mainly due to a shortage of “physical interpreters”, i.e., professional interpreters who come to the hospital to participate in psychological consultations. Indeed, one key element highlighted in our study is the major importance of language barriers for patients with difficulties. Addressing communication barriers for refugees and migrants in healthcare settings has been a major challenge in recent years [29, 30]. A recent scoping review by the WHO European Region examined strategies that have been implemented and evaluated to address communication barriers experienced by refugees and migrants in healthcare, and four main types of strategy were identified: cultural mediation, interpretation, translation of health information, and guidance and training for healthcare providers [30]. In current practice, in France, these strategies are barely used. For instance, our department, although being a referral center, has an only partial and intermittent access to such means. For example, there is no cultural mediation in our department and some other referral centers in France have only access to part-time ethnopsychologists once or twice a week. Lack of cultural mediation and of professional translators has been pointed out as a barrier to tuberculosis care in recent reviews [31–33]. In referral centers in France, interpreting is provided by a complex and costly telephone service which is only used at key moments of hospitalization (e.g., admission, introduction of treatment) but does not allow daily discussions. Moreover, in our department, medical information documents are often only available in French, and at best in English. This is a point on which our department is currently working, as it could be an easy lever to activate. We try to implement a systematic written translation of TB related information for all patients in their own language, using a non-stigmatizing vocabulary and pictures, as described in the literature [34, 35]. We are also currently working on picture-based flipcharts (thus overcoming language barrier and/or illiteracy), that illustrate the importance of remaining in prolonged isolation and taking anti-tuberculosis medications every day.

Language barriers lead to misunderstanding and particularly to misunderstanding of department rules. During the psychological consultation following a conflict situation, the majority of patients (89.5%) declared that their main difficulty was frustration at being forced to stay in hospital. This frustration may have contributed to a strong sense of alienation, which can lead to violence towards healthcare workers, in a context in which medical workplace violence is becoming an alarming and increasing issue worldwide [36–38]. In our study, 9 patients (13.8%) were excluded from the department for disciplinary reasons, because they did not adhere to the department rules, leading to care failure and staff fatigue. Some studies in literature, led in mental health wards, showed a correlation between aggressive behaviors of patients and staff exhaustion [39, 40]. Although caregivers are aware of migrants’ vulnerabilities, they have not received specific training to meet such challenges.

Among those 9 patients, 7 were violent and still needed to be isolated to prevent transmission, so they had to be transferred to another resistant TB referral center to be adequately isolated and cared for by a new medical team with whom they have no history of conflict. Thus, these behavioral issues compromise the good continuity of care.

Because of the slow pace of implementation of specific support for these vulnerable patients with language barriers, other, unfortunately radical, solutions have been discussed. Some cities like New York and London created coercive measures in the 1990s to prevent tuberculosis transmission by detaining patients in isolation rooms in guarded wards [41–43]. In France, there is still a legislative gap: on the one hand, isolation of these patients is a necessary public health measure, while, on the other hand, forced hospitalization is not legally possible because of the respect of individual freedom and the principles of medical ethics. In our study, a patient needed to be guarded by a policeman to prevent him from leaving his hospital room but this deterrent strategy failed because there is no legal support allowing the French authorities to force hospitalization. Indeed, the guard could stay in front of the room, but could not physically restrain the patient to prevent him from leaving. This dilemma is currently discussed by health and ethical authorities in France, around Europe and worldwide. Another option, as suggested by the recent WHO guidelines, is the decentralized (outpatient) DR-TB care. A recent meta-analysis showed that decentralized DR-TB care improved access to care, was more cost-effective than hospital-based care, and could maybe improve treatment outcome [44]. Our study shows that at least 20.0% of our patients left the facilities without medical authorization at least once during hospitalization, and 11 patients (16.9%) did not adhere to respiratory isolation precautions. Long periods of isolation, as observed in our study, may also explain the emergence of these behaviors. Decentralized DR-TB care, in addition to updates in shorter drug-regimens [7–9] might be one option to consider in patients with isolation-induced violence issues [45].

As mentioned, our analysis has the inherent limitations of retrospective single-center studies. First, the figures are probably underestimated. Indeed, even though there was a systematic description of patients’ behavior in nursing records, it was sometimes very brief and not detailed. We can thus suppose that conflict situations could be more frequent and/or at least more complex than what we have reported here. Also, our findings rely on reports, and not on certified facts, which could have led to a reporting bias by the healthcare workers. This nevertheless highlights the most problematic facts from the point of view of these healthcare workers, which remains a relevant point. Thus, we also recall that the study does not allow us to prejudge the origin of conflicts, for which healthcare workers and the institution may also bear a responsibility (i.e., less patience and listening for patients who do not speak the same language). Qualitative data on drivers of inappropriate care situations, from patients’ perspectives, are certainly lacking. Finally, the small number of patients did not permit a multivariate analysis, which could have been useful to take potential confounders into account. This study must certainly be completed by quantitative and qualitative multicenter studies.

## Conclusions

Conflict or inappropriate care situations with healthcare workers concerned more than one-third of patients with MDR/XDR TB in this referral TB center during hospitalization. This study illustrates the urgent need for healthcare systems to address the challenge of a patient-centered approach in the provision of care to MDR/XDR TB patients, for the benefit of both patients and better control of transmission. This highlights in particular the need to reduce the communication barriers (cultural mediation, translation of health information, guidance and training for healthcare providers) and to address either the feasibility of decentralized DR-TB care, or the adaptation of hospital units for long stays.

## Supporting information

S1 Checklist STROBE checklist completed. (DOCX)

S1 Data Complete database. (XLSX)

## References

[1] Global tuberculosis report 2020. [cited 31 Oct 2020]. Available: https://www.who.int/publications-detail-redirect/9789240013131

[2] Global Tuberculosis Report 2021. [cited 7 Nov 2021]. Available: https://www.who.int/teams/global-tuberculosis-programme/tb-reports/global-tuberculosis-report-2021

[3] European Centre for Disease Prevention and Control, WHO Regional Office for Europe. Tuberculosis surveillance and monitoring in Europe 2021–2019 data. Copenhagen: WHO Regional Office for Europe; 2021.

[4] Bernard C, Brossier F, Sougakoff W, Veziris N, Frechet-Jachym M, Metivier N, et al. A surge of MDR and XDR tuberculosis in France among patients born in the Former Soviet Union. Euro Surveill Bull Eur Sur Mal Transm Eur Commun Dis Bull. 2013;18: 20555. doi: 10.2807/1560-7917.es2013.18.33.20555 23968874

[5] Guthmann J.-P, Comolet T, Trébucq A EMC. Pneumologie, 2019, p. 1–16.

[6] Dheda K, Chang KC, Guglielmetti L, Furin J, Schaaf HS, Chesov D, et al. Clinical management of adults and children with multidrug-resistant and extensively drug-resistant tuberculosis. Clin Microbiol Infect. 2017;23: 131–140. doi: 10.1016/j.cmi.2016.10.008 27756712

[7] WHO Consolidated Guidelines on Tuberculosis, Module 4: Treatment—Drug-Resistant Tuberculosis Treatment. [cited 7 Nov 2021]. Available: https://www.who.int/publications-detail-redirect/9789240007048

[8] Nunn AJ, Phillips PPJ, Meredith SK, Chiang C-Y, Conradie F, Dalai D, et al. A Trial of a Shorter Regimen for Rifampin-Resistant Tuberculosis. N Engl J Med. 2019;380: 1201–1213. doi: 10.1056/NEJMoa1811867 30865791

[9] Conradie F, Diacon AH, Ngubane N, Howell P, Everitt D, Crook AM, et al. Treatment of Highly Drug-Resistant Pulmonary Tuberculosis. N Engl J Med. 2020;382: 893–902. doi: 10.1056/NEJMoa1901814 32130813 PMC6955640

[10] World Health Organization. Global tuberculosis report 2018. World Health Organization; 2018. Available: http://apps.who.int/iris/handle/10665/274453

[11] O’Donnell MR, Jarand J, Loveday M, Padayatchi N, Zelnick J, Werner L, et al. High Incidence of Hospital Admissions with Multidrug Resistant and Extensively Drug Resistant Tuberculosis among South African Health Care Workers. Ann Intern Med. 2010;153: 516–522. doi: 10.7326/0003-4819-153-8-201010190-00008 20956708 PMC3074259

[12] Nellums LB, Rustage K, Hargreaves S, Friedland JS. Multidrug-resistant tuberculosis treatment adherence in migrants: a systematic review and meta-analysis. BMC Med. 2018;16. doi: 10.1186/s12916-017-1001-7 29466983 PMC5822608

[13] Nicole S. The public health dimension of the European migrant crisis.: 8.

[14] Zumla A, Abubakar I. Improving access to multi-drug resistant tuberculosis diagnostic and health services for refugees and migrants. BMC Med. 2018;16: 221. doi: 10.1186/s12916-018-1218-0 30497477 PMC6267830

[15] People-centred health care: a policy framework. World Health Organization. Regional Office for the Western Pacific.2007.

[16] HCSP. Tuberculoses à bacilles résistants: diagnostic et prise en charge. Rapp HCSP. Paris: Haut Conseil de la Santé Publique; 2014 Dec. Available: https://www.hcsp.fr/explore.cgi/avisrapportsdomaine?clefr=483

[17] Guglielmetti L, Jaffré J, Bernard C, Brossier F, El Helali N, Chadelat K, et al. Multidisciplinary advisory teams to manage multidrug-resistant tuberculosis: the example of the French Consilium. Int J Tuberc Lung Dis Off J Int Union Tuberc Lung Dis. 2019;23: 1050–1054. doi: 10.5588/ijtld.18.0779 31627768

[18] D’Ambrosio L, Bothamley G, Caminero Luna JA, Duarte R, Guglielmetti L, Muñoz Torrico M, et al. Team approach to manage difficult-to-treat TB cases: Experiences in Europe and beyond. Pulmonology. 2018;24: 132–141. doi: 10.1016/j.rppnen.2017.10.005 29229274

[19] Directive 2008/115/EC of the European Parliament and of the Council of 16 December 2008 on common standards and procedures in Member States for returning illegally staying third-country nationals.

[20] Lafeuille E, Veziris N, Sougakoff W, Roure F, Le Dû D, Dournon N, et al. XDR-tuberculosis in France: Community transmission due to non-compliance with isolation precautions. Médecine Mal Infect. 2016;46: 52–55. doi: 10.1016/j.medmal.2015.12.008 26809373

[21] Ködmön C, Zucs P, Werf MJ van der. Migration-related tuberculosis: epidemiology and characteristics of tuberculosis cases originating outside the European Union and European Economic Area, 2007 to 2013. Eurosurveillance. 2016;21: 30164. doi: 10.2807/1560-7917.ES.2016.21.12.30164 27039665

[22] Hargreaves S, Lönnroth K, Nellums LB, Olaru ID, Nathavitharana RR, Norredam M, et al. Multidrug-resistant tuberculosis and migration to Europe. Clin Microbiol Infect. 2017;23: 141–146. doi: 10.1016/j.cmi.2016.09.009 27665703

[23] Heeren M, Wittmann L, Ehlert U, Schnyder U, Maier T, Müller J. Psychopathology and resident status—comparing asylum seekers, refugees, illegal migrants, labor migrants, and residents. Compr Psychiatry. 2014;55: 818–825. doi: 10.1016/j.comppsych.2014.02.003 24636190

[24] Bustamante LHU, Cerqueira RO, Leclerc E, Brietzke E. Stress, trauma, and posttraumatic stress disorder in migrants: a comprehensive review. Braz J Psychiatry. 2017;40: 220–225. doi: 10.1590/1516-4446-2017-2290 29069252 PMC6900760

[25] Baranyi G, Cassidy M, Fazel S, Priebe S, Mundt AP. Prevalence of Posttraumatic Stress Disorder in Prisoners. Epidemiol Rev. 2018;40: 134–145. doi: 10.1093/epirev/mxx015 29596582 PMC5982805

[26] Zerach G, Shevlin M, Cloitre M, Solomon Z. Complex posttraumatic stress disorder (CPTSD) following captivity: a 24-year longitudinal study. Eur J Psychotraumatology. 2019;10: 1616488. doi: 10.1080/20008198.2019.1616488 31191830 PMC6541897

[27] Baudet T, Saglio-Yatzimirsky M-C. [Migrants’ mental health:invisible wounds]. Rev Prat. 2019;69: 672–675. 31626432

[28] Walker IF, Baral SC, Wei X, Huque R, Khan A, Walley J, et al. Multidrug-resistant tuberculosis treatment programmes insufficiently consider comorbid mental disorders. Int J Tuberc Lung Dis Off J Int Union Tuberc Lung Dis. 2017;21: 603–609. doi: 10.5588/ijtld.17.0135 28482954

[29] McGarry O. What strategies to address communication barriers for refugees and migrants in health care settings have been implemented and evaluated across the WHO European Region?: themed issues on migration and health, IX. 2018. 30484995

[30] Strategy and action plan for refugee and migrant health in the WHO European Region. 7 Dec 2017 [cited 13 Jul 2019]. Available: http://www.euro.who.int/en/health-topics/health-determinants/migration-and-health/publications/2016/strategy-and-action-plan-for-refugee-and-migrant-health-in-the-who-european-region

[31] Barriers and facilitators to the uptake of tuberculosis diagnostic and treatment services by hard-to-reach populations in countries of low and medium tuberculosis incidence: a systematic review of qualitative literature—The Lancet Infectious Diseases. [cited 16 Jan 2022]. Available: https://www.thelancet.com/journals/laninf/article/PIIS1473-3099(16)30531-X/fulltext 10.1016/S1473-3099(16)30531-X 28291721

[32] Lin S, Melendez-Torres GJ. Critical interpretive synthesis of barriers and facilitators to TB treatment in immigrant populations. Trop Med Int Health TM IH. 2017;22: 1206–1222. doi: 10.1111/tmi.12938 28815873

[33] Soedarsono S, Mertaniasih NM, Kusmiati T, Permatasari A, Juliasih NN, Hadi C, et al. Determinant factors for loss to follow-up in drug-resistant tuberculosis patients: the importance of psycho-social and economic aspects. BMC Pulm Med. 2021;21: 360. doi: 10.1186/s12890-021-01735-9 34758794 PMC8579625

[34] Engel N. Towards patient-centred care: analysing TB treatment literacy documents and adherence discourses. Int J Tuberc Lung Dis Off J Int Union Tuberc Lung Dis. 2018;22: 238. doi: 10.5588/ijtld.18.0068 29471898

[35] Brumwell A, Noyes E, Kulkarni S, Lin V, Becerra MC, Yuen CM. A rapid review of treatment literacy materials for tuberculosis patients. Int J Tuberc Lung Dis Off J Int Union Tuberc Lung Dis. 2018;22: 336–341. doi: 10.5588/ijtld.17.0363 29471913

[36] Pompeii LA, Schoenfisch AL, Lipscomb HJ, Dement JM, Smith CD, Upadhyaya M. Physical assault, physical threat, and verbal abuse perpetrated against hospital workers by patients or visitors in six U.S. hospitals. Am J Ind Med. 2015;58: 1194–1204. doi: 10.1002/ajim.22489 26076187

[37] Lepping P, Lanka SV, Turner J, Stanaway SE, Krishna M. Percentage prevalence of patient and visitor violence against staff in high-risk UK medical wards. Clin Med. 2013;13: 543–546. doi: 10.7861/clinmedicine.13-6-543 24298096 PMC5873651

[38] Wang N, Yang S, Zhou X, Hesketh T. Workplace violence and its aftermath among health workers in Zhejiang province: a cross-sectional study. The Lancet. 2017;390: S81. doi: 10.1016/S0140-6736(17)33219-1

[39] Yu JJ, Holbeach E. Aggressive patient behaviours and unplanned nursing staff leave—is there an association? Int J Ment Health Nurs. 2021;30: 1183–1192. doi: 10.1111/inm.12869 33843143

[40] Jalil R, Huber JW, Sixsmith J, Dickens GL. Mental health nurses’ emotions, exposure to patient aggression, attitudes to and use of coercive measures: Cross sectional questionnaire survey. Int J Nurs Stud. 2017;75: 130–138. doi: 10.1016/j.ijnurstu.2017.07.018 28797822

[41] Gasner MR, Maw KL, Feldman GE, Fujiwara PI, Frieden TR. The use of legal action in New York City to ensure treatment of tuberculosis. N Engl J Med. 1999;340: 359–366. doi: 10.1056/NEJM199902043400506 9929527

[42] Lerner BH. Catching patients: tuberculosis and detention in the 1990s. Chest. 1999;115: 236–241. doi: 10.1378/chest.115.1.236 9925090

[43] Coker RJ. National survey of detention and TB. Thorax. 2001 Oct;56(10):818. doi: 10.1136/thorax.56.10.818 11596567 PMC1745929

[44] Ho J, Byrne AL, Linh NN, Jaramillo E, Fox GJ. Decentralized care for multidrug-resistant tuberculosis: a systematic review and meta-analysis. Bull World Health Organ. 2017;95: 584–593. doi: 10.2471/BLT.17.193375 28804170 PMC5537756

[45] Gray AT, Boyles T, Luedtke S, Sossen B, Birjovanu G, Kostkova P, et al. A threat to decentralised care for drug-resistant tuberculosis. Lancet Respir Med. 2020;8: 950–952. doi: 10.1016/S2213-2600(20)30392-1 33007284 PMC7524514

